# Supplementary material for: Excitonic Mott insulator in a Bose-Fermi-Hubbard system of moiré WS2/WSe2 heterobilayer
Source: Nat Commun. 2024 Mar 14;15:2305. doi: 10.1038/s41467-024-46616-x (PMC11258127; doi:10.1038/s41467-024-46616-x)
Supplement: Supplementary file 1 — Supplementary Information [file 41467_2024_46616_MOESM1_ESM.pdf]

Supplementary Information: Excitonic Mott insulator in a Bose-Fermi-Hubbard system of moiré WS_2/WSe_2 heterobilayer

Beini Gao,[1,*] Daniel G. Suárez-Forero*,[1,†] Supratik Sarkar,[1,*] Tsung-Sheng Huang,[1] Deric Session,[1] Mahmoud Jalali Mehrabad,[1] Ruihao Ni,[2] Ming Xie,[3] Pranshoo Upadhyay,[1] Jonathan Vannucci,[1] Sunil Mittal,[1] Kenji Watanabe,[4] Takashi Taniguchi,[4] Atac Imamoglu,[5] You Zhou,[2,6] and Mohammad Hafezi[1,7,‡]

[1] *Joint Quantum Institute (JQI), University of Maryland, College Park, MD 20742, USA*
[2] *Department of Materials Science and Engineering, University of Maryland, College Park, MD 20742, USA*
[3] *Condensed Matter Theory Center, University of Maryland, College Park, MD 20742, USA*
[4] *National Institute for Materials Science, Tsukuba, Japan*
[5] *Institute of Quantum Electronics, ETH Zurich, CH-8093 Zurich, Switzerland*
[6] *Maryland Quantum Materials Center, College Park, Maryland 20742, USA*
[7] *Institute for Theoretical Physics, ETH Zurich, 8093 Zurich, Switzerland*

CONTENTS

1. Device fabrication
2. Optical measurements
3. Electronic filling factor calibration
4. Determination of stacking angle via Second Harmonic Generation
5. Total emitted PL vs. Pump Intensity
6. Pulsed vs. CW excitation
7. Data analysis
8. Modeling X_1 and X_2
9. Diffusion measurements
10. System's spectrum in the purely bosonic limit
11. Reproduction of the observations in Device D2
12. Comparison of U_{ex-ex} and U_{ex-e} with previously reported values
13. Optical signatures of photo-doping effect

Supplementary Note 1. Device fabrication

The WSe_2/WS_2 heterostructure shown in Supplementary Figure 1 was fabricated using a dry-transfer method with a stamp made of a poly(bisphenol A carbonate) (PC) layer on polydimethylsiloxane (PDMS) [1]. All flakes were exfoliated from bulk crystals onto Si/SiO_2 (285 nm) and identified by their optical contrast. The top/bottom gates and TMD contact are made of few-layer graphene. The PC stamp and samples were heated to 60°C during the pick-up steps and released from the stamp to the substrate at 180°C. The PC residue on the device was removed in chloroform followed by a rinse in isopropyl alcohol and ozone clean. Sample transfer was performed in an argon-filled glovebox for improved interface quality. The electrodes consist of 3.5 nm of chromium and 70 nm of gold. They were fabricated using standard electron-beam lithography techniques and thermal evaporation.

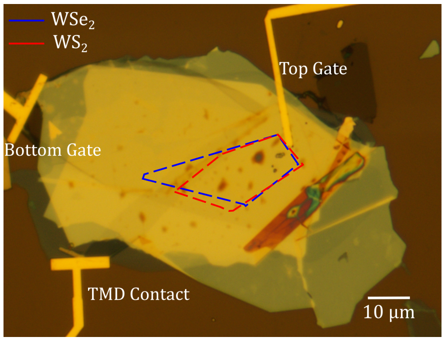

Supplementary Figure 1. Microscope image of the WS_2/WSe_2 heterostructure device.

Supplementary Note 2. Optical measurements

The sample is kept in a dilution refrigerator at a temperature of 3.5K. For photoluminescence measurements, we use a confocal microscopy setup with an objective of magnification 70× and numerical aperture NA = 0.82. Our pumping source is a pulsed Ti:Sapphire laser tuned at 720nm (1.722eV), with a pulse duration of 100fs and a repetition rate of ∼ 80 MHz. A half-waveplate placed before a polarizing beam-splitter is rotated to control the pump power. Additionally, an optical chopper system at 800Hz is used to prevent sample heating while having a high pump intensity. A 750nm long-pass filter was used to block the residual pump laser before collecting the PL emission in a spectrometer equipped with a 300 grooves per mm diffraction grating and a CCD camera. The schematic of the setup is shown in Supplementary Figure 2.

For the diffusion measurements, we used a continuous-wave (CW) Ti:Sapphire laser tuned at 708nm. The rest of the optical measurement setup was similar. By applying a spatial filter to the reconstructed image of the diffusion pattern, we obtain spectral and spatial data of the exciton emission. This allows to image the spatial diffusion of each spectral component and extract the diffusion length.

Supplementary Note 3. Electronic filling factor calibration

The electronic filling factor (ν_e) can be estimated by combining the information about the crystalline structure of the bilayer device and a parallel capacitor model, as follows:

Determination of moiré density $\mathbf{n_0}$: In a purely fermionic Mott insulating state, the heterostructure will host one electron per moiré unit cell. The charge density in this case is given by $\mathrm{n_0}$, and it can be directly determined by the moiré periodicity through the relationship $\mathrm{n_0} = 1/({L_M}^2 \sin\pi/3)$. Here, $L_M = a/\sqrt{\delta^2+\theta^2}$ is the size of the moiré superlattice, $\delta = (a-a')/a \approx 4\%$ is the lattice mismatch between WSe_2 ($a = 0.328$nm) and WS_2 ($a' = 0.315$nm), and θ is the twist angle between the two layers. Assuming $0^\circ \leq \theta \leq 1^\circ$, we obtain $1.72 \times 10^{12}\mathrm{cm}^{-2} \leq \mathrm{n_0} \leq 2.04 \times 10^{12}\mathrm{cm}^{-2}$.

Determination of electron density $\mathbf{n_e}$: From a parallel capacitor model, we can deduce the expression for the electron density (n_e) in the bilayer. For a dual gate device, n_e is given by:

$$n_\mathrm{e} = \frac{\epsilon_\mathrm{r}\epsilon_0\Delta V_\mathrm{g}}{d_\mathrm{t}} + \frac{\epsilon_\mathrm{r}\epsilon_0\Delta V_\mathrm{g}}{d_\mathrm{b}},$$

where V_g is the symmetrically applied gate voltage and $d_\mathrm{t} \approx d_\mathrm{b} \approx 40$nm are the thicknesses of the top and bottom hBN dielectrics, respectively. They are determined from the optical contrast of the hBN flakes under the microscope. ϵ_0 is the vacuum permittivity, and $\epsilon_r \approx 3$ is the relative permittivity of hBN [2]. Having the moiré density n_0 and the electron density n_e, the electronic (fermionic) filling factor can be expressed as $\nu_\mathrm{e} = n_\mathrm{e}/n_0$. From this model, we deduce that the gate voltage at which the electronic Mott insulator is established lies in the range 2.65 V$< V_\mathrm{g} <$ 3.04 V. Where we are taking into account that the neutral region extends up to $V_\mathrm{g} = 0.58$ V. This estimation is in good agreement with our experimental data for reflectivity and PL. The results, displayed in Supplementary Figure 3 show a consistent change in the optical response of the device at $V_\mathrm{g} = 2.98$ V. For the reflectivity (panel a), we observe a shift in the intralayer exciton energy. For the PL (panel b), a corresponding energy transition is observed for the interlayer exciton.

Although this calibration holds for both the hole-doped and the electron-doped sides, Supplementary Figure 3b shows an asymmetric behavior of the exciton interaction with the sign of the doping: the energy gap at $\nu_e = 1$ on the electron doping side is larger than the one in the hole doping side. This is in agreement with theoretical predictions [3] and experimental ob-

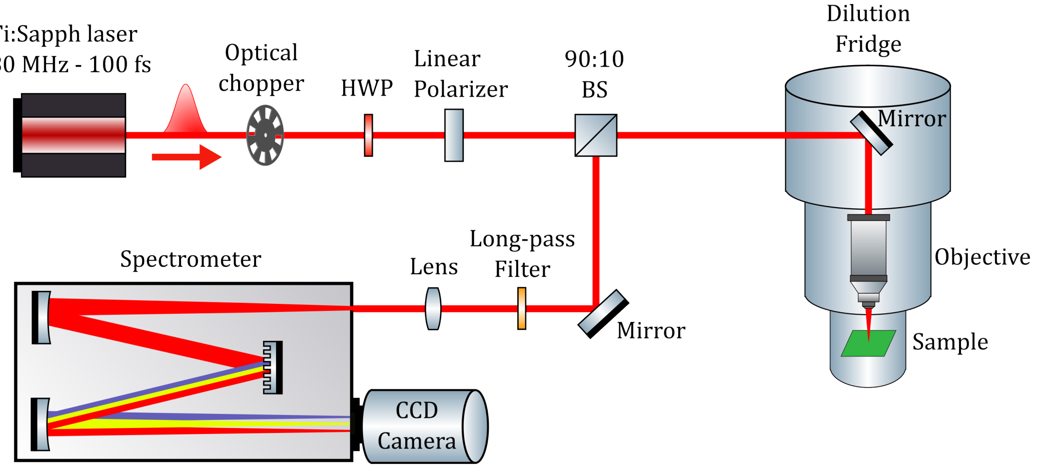

Supplementary Figure 2. Schematic of the experimental setup. HWP=Half-wave plate, BS=Beam splitter

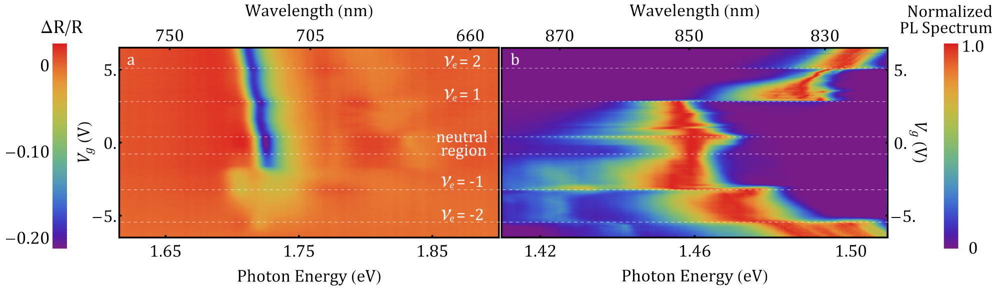

Supplementary Figure 3. Reflection and photoluminescence spectra of our WS_2/WSe_2 heterobilayer device.

servations [4, 5] that show a reduced value of the interaction strength due to the fact that holes and excitons reside in different high symmetry points of the moiré lattice. This observation indicates that electron doping is more suitable for the study of Fermi-Bose hybrid correlated states.

Supplementary Note 4. Determination of stacking angle via Second Harmonic Generation

We performed Second Harmonic Generation (SHG) measurements on our device in order to determine the stacking angle of the component monolayers. This is important because the system's properties vary for R-stacking and H-stacking. Using a 100 fs excitation at 850 nm with variable linear polarization, we are able to reconstruct the expected behavior of the SHG angular dependence for a crystalline structure with inversion symmetry breaking (Supplementary Figure 4a). We first identify spots on the sample with bilayer and monolayer regions by using the PL emission for the characterization (panel b). After identifying each spot, we proceed to measure the SHG spectrum while keeping the fs laser power constant at $\sim 150\mu W$. As demonstrated in ref. [6] and the Supplementary Material of ref. [7], the stacking angle can generate a constructive (destructive) interference in

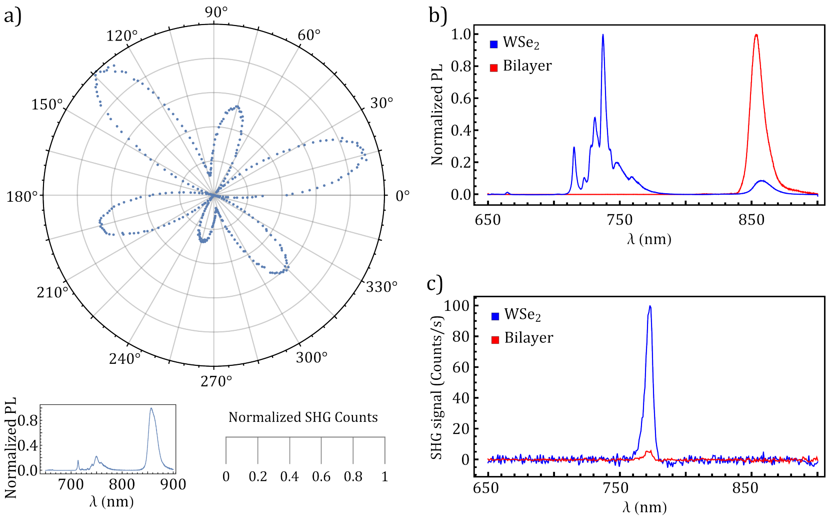

Supplementary Figure 4. SHG sample characterization for the determination of the stacking angle. a) SHG angular dependence from a representative spot in the sample, characterized by the PL spectrum (inset). b) Characteristic PL emission spectra of spots on the sample with WSe_2 monolayer (blue) or WSe_2/WS_2 bilayer (red). c) SHG corresponding to the two spots of panel b. The strong suppression of the SHG emission efficiency indicates that the sample is stacked at an angle of 60°.

the SHG spectrum for 0° (60°) stacking. The strong suppression in the SHG efficiency observed in panel c, allows us to conclude that our sample corresponds to a bilayer with a 60° stacking angle (AB or H stacking).

Supplementary Note 5. Total emitted PL vs. Pump Intensity

Due to the saturability of real semiconductor materials upon an intense optical pump, the laser intensity I is not a suitable parameter to estimate the total exciton density in the bilayer. One can corroborate this from Supplementary Figure 5, by noticing that the emitted PL power does not follow a linear trend with increasing I. A more suitable quantity to monitor the changes in the excitonic density is the total emitted PL power. This quantity can be considered proportional to the total number of excitons formed in the structure, with the proviso that the radiative decay of the excitons does not change considerably within the phase space determined by V_g and I. For this reason, Figures 2 and 3 of the main text display the total PL power instead of the pump intensity. For simplicity, all the PL powers presented in the main text are normalized to a factor 10^6.

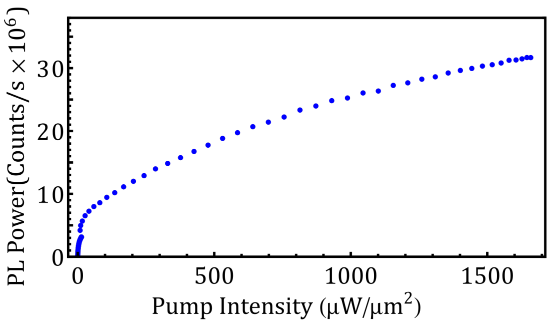

Supplementary Figure 5. Pump intensity dependent total PL power at $V_g = 0V$

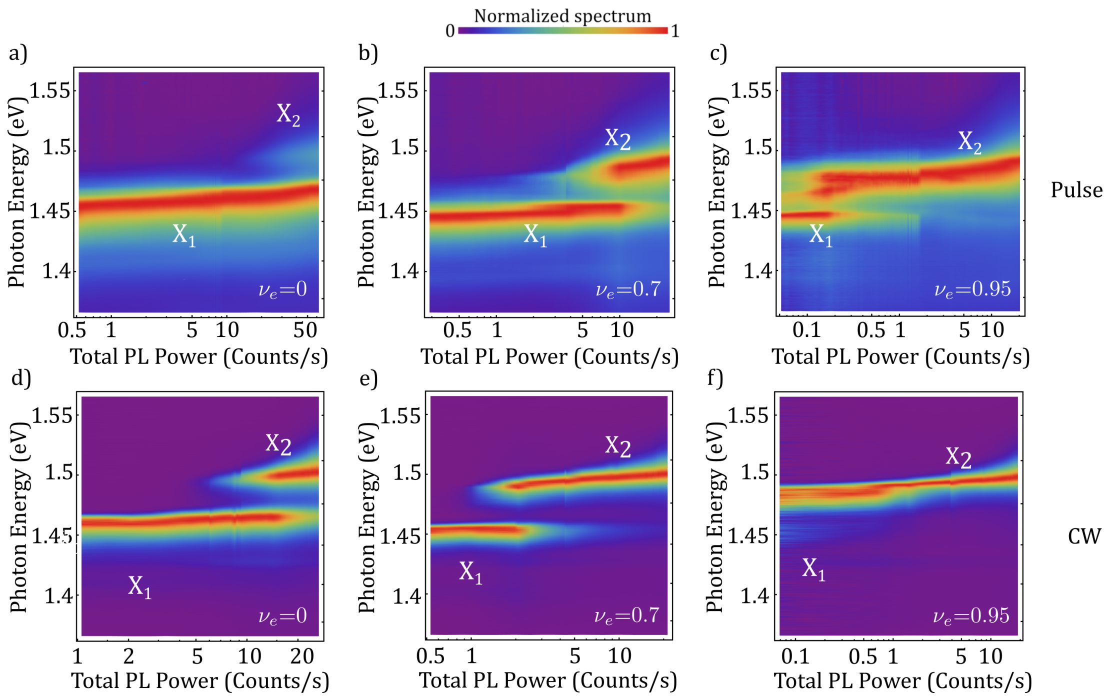

Supplementary Figure 6. Normalized PL spectrum as a function of the total collected PL power for three different electronic filling factors under pulsed (a-c) and CW (d-f) excitations. The peaks associated with single (X1) and double (X2) occupancies are indicated on each panel. The qualitative behavior remains unaltered under both excitation regimes.

Supplementary Note 6. Pulsed vs. CW excitation

Although we performed experiments on both CW and pulsed regimes, the pulsed excitation has the important capability of reaching densely populated states while avoiding thermal effects, since the same populations can be achieved with much lower average power. Since Figs. 1, 2, and 5 of the main text present data collected in a pulsed excitation regime, it is important to verify that the high instant powers of the pulsed excitation are not inducing more complex nonlinear effects. This verification is done by comparing the data acquired in both excitation regimes. We use a Ti:Sapphire CW laser and perform the experiment in the same conditions. As shown in Supplementary Figure 6, the qualitative behavior of the system is the same as in Supplementary Figure 2 of the main text: at low gate voltage only the single occupancy states are detected, and for increasing exciton density a secondary peak indicates the creation of double occupancies in the moiré lattice. The gate voltage dependence is also consistent: as one populates the lattice with electrons, the secondary peak becomes visible at lower gate voltages.

Supplementary Note 7. Data analysis

To obtain relevant information about the collected spectra, we use a fitting routine. This procedure allows us to obtain the PL emission central energy, linewidth, and integrated

PL power of each exciton line (X_1 and X_2). First, the PL spectra are processed by removing background noise and applying a low-pass Butterworth filter. After that, we extract the peaks from each spectrum by imposing constraints on the energy range, linewidth, prominence, and relative amplitude to the noise level. By setting a tolerance to the numerical error of the obtained values of intensity and energy of each peak, we fit each spectrum to a multi-Lorentzian distribution. Supplementary Figure 7 shows a representative spectrum with the corresponding fitting using two Lorentzian distributions. From the fitting functions, we extract the central energies of X_1 and X_2 and integrate them over the obtained distributions to get the individual PL power. The Inset of Supplementary Figure 7 shows a wide PL spectrum at low and high powers. At low power, we only observe X_1, while we see both X_1 and X_2 at high pump power. The process of creation of interlayer excitons is so efficient, that no PL is detected from intralayer excitons or trions. Hence, we only focus on the spectrum around the interlayer excitons for data analysis.

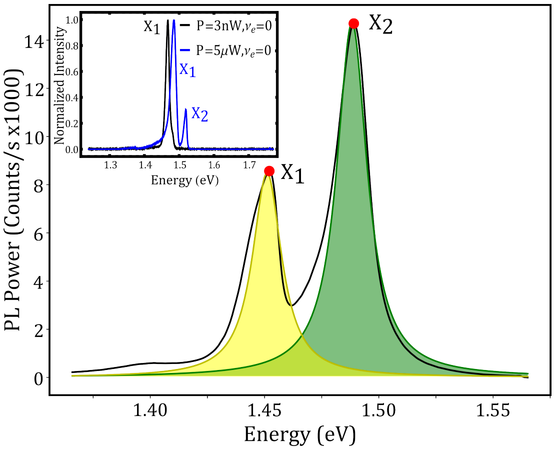

Supplementary Figure 7. A typical PL spectrum (V_g = 2.1 V and I = 2226 μW/μm^2) with the fitting functions obtained from the numerical method. Inset shows a wide PL spectrum at low and high pump powers with no WSe_2 intralayer excitons or trions.

Supplementary Note 8. Modeling X_1 and X_2

To obtain further insights into the physical mechanism dominating the system dynamics, we make a theoretical analysis using a 2-band model, as mentioned in the main text. Our goal is to deduce an analytical expression for the observed saturation behavior of the PL power from the X_1 population. This is achieved by considering the steady state of the system in a continuous-wave excitation regime. A population of electron-hole pairs $n_{\rm eh}$ is created by a Rabi-driving $\Omega(t)$. The charged plasma cascades down and relaxes to create n_1 singly occupied moiré sites (X_1) and n_2 doubly occupied sites (X_2). The formation of those states depends on the relaxation rate of the plasma ($\Gamma_{\rm relax}$), the maximum number of available lattice sites ($n_{\rm max}(\nu_{\rm e})$), and a β factor, which determines the probability of the plasma to decay into the X_1 state. Under these conditions, the system's dynamics can be described by the set of equations:

$$\frac{{\rm d}n_{\rm eh}}{{\rm d}t} = \eta\,|\Omega(t)|^2 - \Gamma_{\rm relax} n_{\rm eh} \tag{1a}$$

$$\frac{{\rm d}n_1}{{\rm d}t} = \Gamma_{\rm relax}\beta\frac{n_{\rm max}(\nu_e) - n_1}{n_{\rm max}(\nu_e)} n_{\rm eh} - \Gamma_1 n_1 \tag{1b}$$

$$\frac{{\rm d}n_2}{{\rm d}t} = \Gamma_{\rm relax}\left(1 - \beta\frac{n_{\rm max}(\nu_e) - n_1}{n_{\rm max}(\nu_e)}\right) n_{\rm eh} - \Gamma_2 n_2 \tag{1c}$$

where η is the efficiency of the optical generation of electron-hole pairs and Γ_1 (Γ_2) is the decay rate of the excitonic states in a singly (doubly) occupied site. In a steady state condition, from Eq. 1b, we can obtain an analytical expression for n_1:

$$n_1 = \frac{\Gamma_{\rm relax}\beta n_{\rm eh}}{\Gamma_1 + \Gamma_{\rm relax}\beta\frac{n_{\rm eh}}{n_{\rm max}}} \tag{2}$$

In this regime n_{eh} reaches an asymptotic value $n_{\mathrm{eh}} = \frac{\eta}{\Gamma_{\mathrm{relax}}} |\Omega|^2$. To associate this expression with our experimental data, we take into account that $n_{\mathrm{eh}}\Gamma_{\mathrm{relax}}$ is proportional to the total PL power and n_1 is proportional to the PL power (P_1) from the X_1 exciton band. After substituting variables we can conclude that the saturation behavior of X_1 should follow the functional form:

$$P_1 = \mathrm{P}_1^{\max} \frac{\mathrm{P}}{\mathrm{P} + \mathrm{P}_{\mathrm{sat}}} \quad (3)$$

where $P_1^{\max} = n_{\max}$ is proportional to the PL power from X_1 in an ideal Mott insulating state and $P_{\mathrm{sat}} = \Gamma_1 n_{\max}/\beta$ determines the total PL emission at which X_1 saturates. This expression is used as a fitting model for the PL power from the X_1 states upon increasing total PL power, and provides a mathematical tool to estimate the bosonic occupation of the moiré lattice. From this theoretical treatment, one can notice that the bosonic Mott insulating states are reached asymptotically. This means that P_1 can be arbitrarily close to $P_1^{\max}$ upon a high enough total PL power. In other words, the line that determines the Mott insulating phases in Figs. 1c, 2d, and 3d of the main text is mathematically not achievable. For this reason, the intensity denoted as I^* in the referred figures, indicates the required pump intensity to drive the system into a state with a total population arbitrarily close to the lattice complete saturation.

Supplementary Note 9. Diffusion measurements

For the diffusion measurements, we use a continuous-wave laser tuned at 708 nm to create a steady population of excitons. By imaging the diffusion pattern with spectral resolution, we are capable of monitoring the diffusion properties of the X_1 and X_2 populations. We study the dependence of the diffusion length for each population as a function of V_{g} and I. To improve the spatial resolution, the image of the diffusion pattern is magnified 200 times. We spectrally resolve the signals emitted from X_1 and X_2. Next, we fit each spatial diffusion profile to a function of the form $A \exp{(-x/L_{\mathrm{X}_i})} + b$, where A is the PL power under the pumping laser spot, x is the propagation distance, L_{X_i} is the diffusion length of X_i and b is an offset that accounts for the base noise level. Supplementary Figure 8 (a-b) shows the obtained power dependence for L_{X_1} at $V_{\mathrm{g}} = 1.7$ V and $V_{\mathrm{g}} = 2.38$ V. We observe that L_{X_1} increases with pump power at 1.7 V but decreases at 2.38 V. This inversion in the power dependence accounts for the formation of incompressible excitonic states. It is worth mentioning that the scale of the I axis is not linear, because the power was modified by changing the polarization of the pump laser with a half-wave plate. Panel c shows the fitting subroutine used to extract the diffusion length of X_1. Panel d shows the measured value of L_{X_1} for a range of V_{g} to highlight the inversion of the intensity dependence. The same analysis was performed for the diffusion of the X_2 states. The data (Supplementary Figure 9a) shows a shorter diffusion length, which makes it cumbersome to detect changes in L_{X2} due to the diffraction limit. However, in contrast to L_{X1}, the L_{X2} increases with increasing V_{g}, except for the reduction at $\nu_{\mathrm{e}} \sim 1$, which can be attributed to the behavior of a population of excitons in presence of a fermionic Mott insulating state. We finally analyze the diffusion data with no spectral resolution. For $\nu_e < 1$, one can observe that the suppression of the diffusion takes place for lower V_{g} upon increasing pump intensity; a consequence of the bosonic saturation of the lattice due to the optical pump.

Delving deeper into the spatial diffusion, we identify a transfer of population from X_2 to X_1 as the excitons move away from the pump region. As observed in Supplementary Figure 10, for high pump power, the population of X_1 away from the excitation spot is larger than under it, a manifestation of the mentioned effect. At the excitation spot, the high exciton density leads

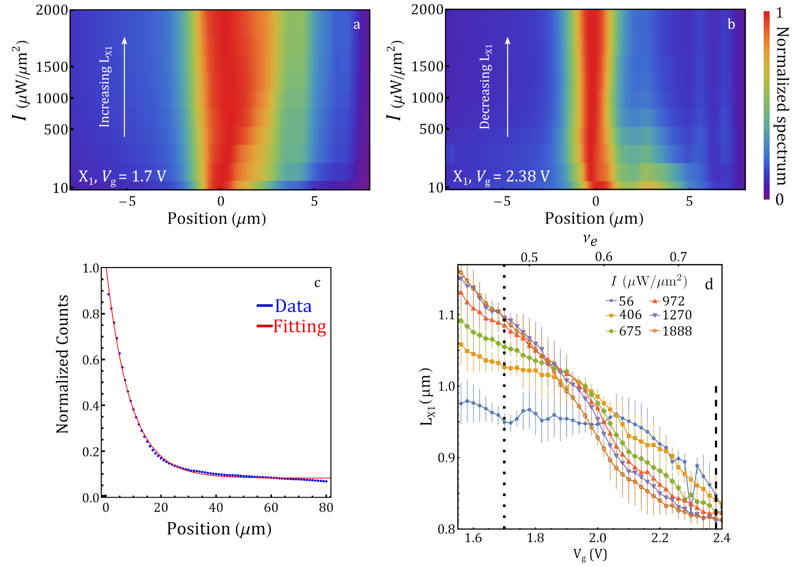

Supplementary Figure 8. (a-b) Pump intensity dependence of the X_1 spatial diffusion for $\nu_e \sim 0.47$ ($V_g = 1.7V$) (a) and $\nu_e \sim 0.75$ ($V_g = 2.38V$) (b). The panels show the inversion of the intensity dependence: from a diluted gas of bosons in panel a to a bosonic Mott insulator in panel b. (c) Exponential decay fitting of a typical X_1 diffusion pattern. (d) L_{X1} for a reduced range of V_g to highlight the power dependence inversion. The error bars represent the standard errors for the diffusion length estimated from the exponential fitting.

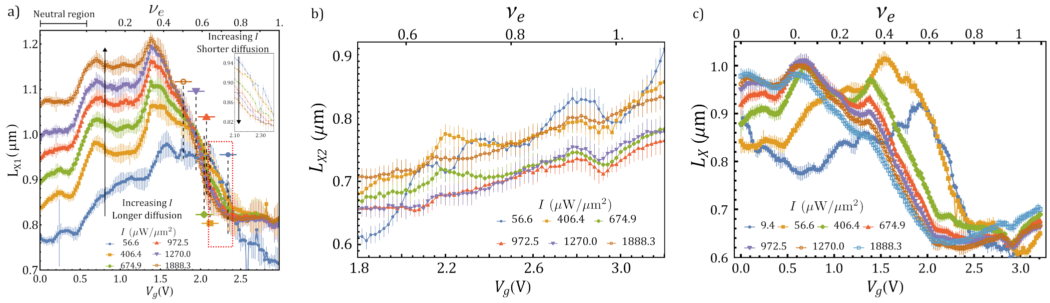

Supplementary Figure 9. (a) L_{X1} as a function of V_g for different pump intensities. In this panel, we include dashed lines corresponding to the crossing gate voltage at each power. They are a guide for the eye of where the incompressibility is expected to manifest at each power. (b)L_{X2} as a function of the gate voltage for a range of ν_e and for different I (c) L_X as a function of the gate voltage for a range of ν_e and different I. The error bars represent the standard errors for the diffusion length estimated from the exponential fitting.

to the formation of a localized Mott insulating state and therefore a large population of X_2. However, as the distance from the pumping region increases, part of the population transfers from X_2 to X_1. Notice how, for high excitation intensity, the X_1 signal becomes stronger away from the injection spot than under it. In this case, the X_2 population is acting as a reservoir for X_1 along the diffusion path. Importantly, this behavior does not change the interpreta-

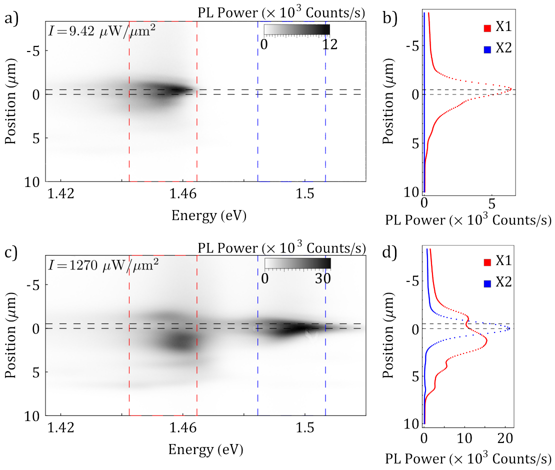

Supplementary Figure 10. Diffusion of the system for $\nu_e = 0$ and two different pumping intensities. a) 9.42 μW/μm^2 and c) 1270 μW/μm^2. The right panels (b and d) show the horizontally integrated intensity. The transfer of population from X_2 to X_1 becomes evident in panel d, where the X_1 intensity is larger outside the pumping spot due to a large reservoir of X_2 excitons that decay into X_1 as they propagate.

tion of our results, but constitutes proof of the robustness of the effect; the population transfer from X_2 to X_1 would favor an apparently longer propagation constant for X_1, which, at most, would hinder the measured suppression of the diffusion.

Supplementary Note 10. System's spectrum in the purely bosonic limit

In the absence of doping electrons, the PL spectrum displays a particular behavior for increasing excitonic population (Supplementary Figure 3a of main text): a blueshift at low occupancy and the emergence of a PL peak from a gapped state after a threshold PL power. In this "reduced" system, where the population is fully bosonic, we can model the system and account for the blueshift and the spectral gap by using the following Lindbladian master equation:

$$\partial_t \hat{\rho} = -i[\hat{H}, \hat{\rho}] + \sum_n \hat{\mathcal{L}}_n[\hat{\rho}],$$

where $\hat{H}$ corresponds to the Bose-Hubbard Hamiltonian:

$$\hat{H} = \omega_{\mathrm{x}} \hat{x}^\dagger \hat{x} + \frac{U_{\mathrm{ex-ex}}}{2} \hat{x}^\dagger \hat{x}^\dagger \hat{x} \hat{x},$$

and the operators $\hat{x}(\hat{x}^\dagger)$ stand for the annihilation (creation) of an exciton particle, ω_{x} is their energy, and $U_{\mathrm{ex-ex}}$ is the on-site particle repulsion interaction energy. We consider two Lindbladian terms to take into account the laser pump and exciton loss. Specifically, the operator for channel n is:

$$\hat{\mathcal{L}}_n[\hat{\rho}] = \hat{C}_n \hat{\rho} \hat{C}_n^\dagger - \frac{1}{2}\{\hat{C}_n^\dagger \hat{C}_n, \hat{\rho}\},$$

that includes the jump operators accounting for two incoherent processes: laser pump and exciton losses, with operators $\hat{C}_p$ and $\hat{C}_l$, respectively:

$$\hat{C}_p = \sqrt{\Gamma_p} \hat{x}^\dagger, \qquad \hat{C}_l = \sqrt{\Gamma_l} \hat{x}.$$

Using the quantum regression theorem for the expected value $\langle \hat{x}^\dagger \hat{x} \rangle$, we calculate the spectral function and extract the central energy of the main peak. Given the set of theory data, we perform a routine for fitting the experimental results, obtaining the set:

$$U_{\mathrm{ex-ex}} \simeq 32.4 \text{ meV}, \qquad \Gamma_l \simeq 10.1 \text{ meV}, \qquad \omega_{\mathrm{x}} \simeq 1460 \text{ meV}$$

The results, displayed in Supplementary Figure 11 show a very good agreement with the experimental results. This figure provides important insights into the nature of $U_{\mathrm{ex-ex}}$. It shows that the on-site exciton repulsion leads to a spectral blueshift even before the system reaches an insulating state. Importantly, such a master equation cannot explain the intensity-dependent behavior of X_1. A unified model including higher-order jump operators (and hence more fitting parameters) may be required. We

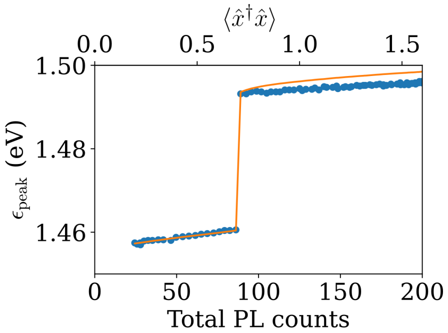

Supplementary Figure 11. Continuous line: Main peak position of the calculated spectral function for a Bose-Hubbard model under the Master equation formalism. The blue circles correspond to the obtained experimental data (from Fig. 3a of the main text) for the PL power dependence of the spectrum at $\nu_e = 0$.

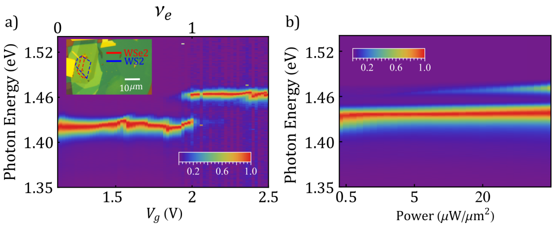

Supplementary Figure 12. (a) Gate voltage-dependent normalized PL in device D2 for a pump intensity of 0.048 μW/μm^2. The splitting between X_1 and X_2 (U_{ex-e}) is 34 meV. Inset shows an optical image of device D2.

(b) Power-dependent normalized PL in device D2 at $\nu_e = 0$. The splitting between X_1 and X_2 (U_{ex-ex}) is 35 meV.

anticipate this master equation description and the rate equations presented in section VIII being simplifications of a unified theory, which needs further investigation.

Supplementary Note 11. Reproduction of the observations in Device D2

Further verification of our experimental results is done by fabricating a second identical

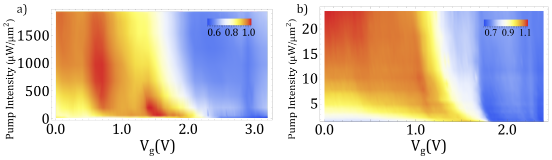

Supplementary Figure 13. Power and gate voltage-dependent diffusion length of X_{tot} in device D1 (same as the device in the main text) and device D2. The difference in the magnitude of the power range is because the exciton lifetime of D2 is about 30 times longer than D1.

device (D2), whose picture can be observed in the inset of Supplementary Figure 12a. We perform electron doping-dependent and power-dependent PL using a CW source. The results, displayed in Supplementary Figure 12a-b are in agreement with the observations reported in the main text.

We also measured the diffusion length in the new device and compared the results with the ones from device D1. In Supplementary Figure 13, we present the diffusion length in devices D1 (same as the one in the main text) and D2 when varying both the electronic filling factor and pump intensity. As it can be observed, in both devices there is a range of gate voltages for which increasing power brings a reduction of the diffusion length, confirming the formation of excitonic correlated states in both structures.

Supplementary Figure 14 presents the time-resolved PL in D2 for different powers and doping levels. Panel d shows a noticeable decrease in the lifetime with increasing gate voltage (electron doping). However, with increasing power, we observe no significant change in lifetime but only the emergence of a fast-decaying population. The existence of two decay times has been reported in both multilayer and monolayer TMD systems [8]. In this device, we detect a fast-decaying population two orders of magnitude lower than the population with long decay times.

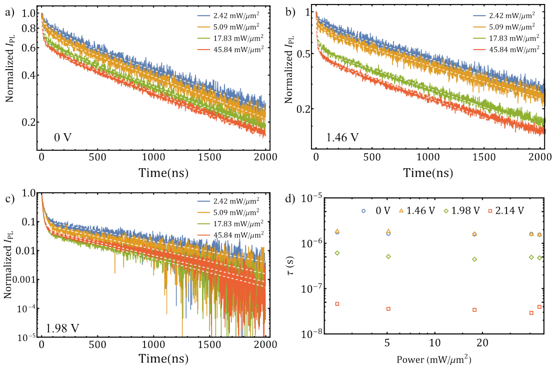

Supplementary Figure 14. Time-dependent PL normalized at t = 0 for different peak powers at 0V (a), 1.46V (b), and 1.98V (c). For this measurement, we use a pulsed laser with a 500-kHz repetition rate and 100 ps "on" time. The power we quoted is the peak power. Dashed white lines correspond to double exponential fits. (d) Long lifetime values at different powers and different gate voltages.

TABLE I. Compilation of reported values of the interaction energies U_{ex-e} and U_{ex-ex} in the literature.

	U_{ex-e} (meV)	U_{ex-ex} (meV)	$\frac{U_{ex-e}}{U_{ex-ex}}$
Device D1	27	32	0.84
Device D2	34	35	1
Park *et al.* (R stack) [9]	-	30-37	-
Lian *et al.* (H stack) [5]	41	32	1.28
Lian *et al.* (R stack) [5]	17	44	0.39
Xiong *et al.* (H stack) [4]	35	15	2.3

Supplementary Note 12. Comparison of U_{ex-ex} and U_{ex-e} with previously reported values

The exciton-exciton interaction energy U_{ex-ex} and exciton-electron interaction energy U_{ex-e} have been recently reported in the literature. Table I compiles them along with the values we measured in our devices. It is important to note the variations seen in the values of interaction energies even in similar heterostructure architectures. Although most of the interaction energies reported are in the range $\sim 15-44$ meV, no clear trend has been observed in experimentally reported values of U_{ex-e}/U_{ex-ex}.

Supplementary Figure 15 shows how we extracted values of interaction energies from the PL spectra. The energy gap between X_1 and X_2 at low power and $\nu_e = 1$ gives us the exciton-electron interaction energy U_{ex-e}, and the energy gap between X_1 and X_2 at lowest power for which X_2 appears even at $\nu_e = 0$ gives us the exciton-exciton interaction energy U_{ex-ex}.

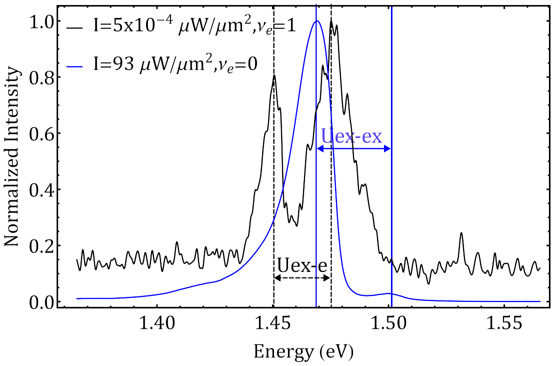

Supplementary Figure 15. PL spectrum of device D1 showing $U_{ex-e} \sim 27$ meV and $U_{ex-ex} \sim 32$ meV.

Supplementary Note 13. Optical signatures of photo-doping effect

Under the strong pumping power regime, one may expect the existence of a photodoping effect in the system. To alleviate this concern, we perform pump power-dependent measurements under off-resonant and on-resonant cases, which show the existence of a photodoping effect, and no photo-doping effect in the system, respectively. Supplementary Figure 16a presents the

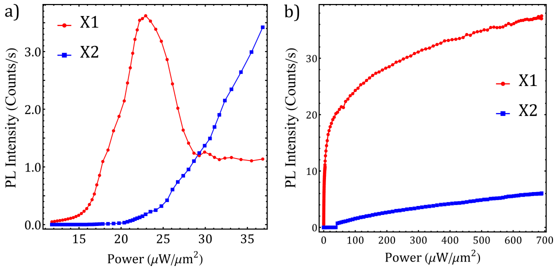

Supplementary Figure 16. Evolution of PL intensity for X_1 (red) and X_2 (blue) as a function of the total excitation intensity for two different excitation regimes: a) device D2 pumped with an out-of-resonant CW laser (633nm) and b) device D1 pumped resonantly (720nm). Due to the photo-doping effect, the intensity of X_1 reduces after a threshold power, in contrast to the resonant excitation, where the PL intensity increases monotonically. In both panels $\nu_e = 0.3$. Like in the main text, the presented counts are divided by a factor 10^6.

X_1 and X_2 energy evolution of device D2 under a non-resonant pump (633 nm), where we can observe a strong photo-doping effect - a reduction of X_1 intensity as a function of pumping power, consistent to the gate voltage-dependent PL spectrum at low power (Fig. 2e in the main text). However, while using the resonant pump (720 nm) for D1, as indicated in panel b, the X_1 emission monotonically increases for the whole range of pump power, an observation confirms that no photo-doping effect exists in the system.

---

* These authors contributed equally to this work

† dsuarezf@umd.edu

‡ hafezi@umd.edu

[1] P. J. Zomer, M. H. D. Guimarães, J. C. Brant, N. Tombros, and B. J. van Wees, Applied Physics Letters **105**, 013101 (2014).

[2] H. C. P. Movva, B. Fallahazad, K. Kim, S. Larentis, T. Taniguchi, K. Watanabe, S. K. Banerjee, and E. Tutuc, Phys. Rev. Lett. **118**, 247701 (2017).

[3] M. H. Naik, E. C. Regan, Z. Zhang, Y. H. Chan, Z. Li, D. Wang, Y. Yoon, C. S. Ong, W. Zhao, S. Zhao, M. I. B. Utama, B. Gao, X. Wei, M. Sayyad, K. Yumigeta, K. Watanabe, T. Taniguchi, S. Tongay, F. H. da Jornada, F. Wang, and S. G. Louie, Nature 2022 609:7925 **609**, 52 (2022).

[4] R. Xiong, J. H. Nie, S. L. Brantly, P. Hays, R. Sailus, K. Watanabe, T. Taniguchi, S. Tongay, and C. Jin, Science **380**, 860 (2023).

[5] Z. Lian, Y. Meng, L. Ma, I. Maity, L. Yan, Q. Wu, X. Huang, D. Chen, X. Chen, X. Chen, M. Blei, T. Taniguchi, K. Watanabe, S. Tongay, J. Lischner, Y.-T. Cui, and S.-F. Shi, (2023), arXiv:2308.10799.

[6] Y. Wang, J. Xiao, S. Yang, Y. Wang, and X. Zhang, Optical Materials Express, Vol. 9, Issue 3, pp. 1136-1149 **9**, 1136 (2019).

[7] C. Jin, Z. Tao, T. Li, Y. Xu, Y. Tang, J. Zhu, S. Liu, K. Watanabe, T. Taniguchi, J. C. Hone, L. Fu, J. Shan, and K. F. Mak, Nature Materials 2021 20:7 **20**, 940 (2021), arXiv:2007.12068.

[8] Y. Jiang, S. Chen, W. Zheng, B. Zheng, and A. Pan, Light: Science & Applications 2021 10:1 **10**, 1 (2021).

[9] H. Park, J. Zhu, X. Wang, Y. Wang, W. Holtzmann, T. Taniguchi, K. Watanabe, J. Yan, L. Fu, T. Cao, D. Xiao, D. R. Gamelin, H. Yu, W. Yao, and X. Xu, Nature Physics 2023 19:9 **19**, 1286 (2023).
